# Lansoprazole-induced osteoporosis via the IP3R- and SOCE-mediated calcium signaling pathways

**DOI:** 10.1186/s10020-022-00448-x

**Published:** 2022-02-19

**Authors:** Ziping Cheng, Yangjie Liu, Mengyuan Ma, Shiyu Sun, Zengqing Ma, Yu Wang, Liyuan Yu, Xuping Qian, Luning Sun, Xuehui Zhang, Yun Liu, Yongqing Wang

**Affiliations:** 1 Research Division of Clinical Pharmacology, The First Affiliated Hospital of Nanjing Medical University and Jiangsu Province Hospital, 300 Guangzhou Road, Nanjing, 210009 China; 2 Department of Pharmacy, Jiangsu Shengze Hospital, Nanjing Medical University, Suzhou, China; 3 Department of Pharmacy, Nanjing Medical University, Nanjing, China; 4 Department of Geriatrics Endocrinology, The First Affiliated Hospital of Nanjing Medical University, Jiangsu Province Hospital, 300 Guangzhou Road, Nanjing, 210029 China

**Keywords:** Lansoprazole, IP3R, SOCE, ER stress, Calcium overload, Osteoporosis

## Abstract

**Background:**

Many clinical studies have shown a correlation between proton pump inhibitors (PPIs) and osteoporosis or fractures. The purpose of this study was to establish a murine model of chronic oral PPI administration to verify whether PPIs caused bone metabolic impairment and investigate the relevant molecular mechanism underlying the effects of PPIs on MC3T3-E1 murine osteoblasts.

**Methods:**

A lansoprazole-induced bone loss model was used to investigate the damaging effects of PPIs. In vivo, immunohistochemistry, Hematoxylin–Eosin (HE) staining, micro-CT analysis, and blood biochemical analyses were used to evaluate the effect of lansoprazole on bone injury in mice. In vitro, the effects of lansoprazole and related signaling pathways in MC3T3-E1 cells were investigated by CCK-8 assays, EdU assays, flow cytometry, laser confocal microscopy, patch clamping, reverse transcription-quantitative polymerase chain reaction and Western blotting.

**Results:**

After 6 months of lansoprazole gavage in ICR mice, the micro-CT results showed that compared with that in the vehicle group, the bone mineral density (BMD) in the high-dose group was significantly decreased (*P* < 0.05), and the bone microarchitecture gradually degraded. Biochemical analysis of bone serum showed that blood calcium and phosphorus were both decreased (*P* < 0.01). We found that long-term administration of lansoprazole impaired skeletal function in mice. In vitro, we found that lansoprazole (LPZ) could cause calcium overload in MC3T3-E1 cells leading to apoptosis, and 2-APB, an inhibitor of IP3R calcium release channel and SOCE pathway, effectively blocked increase in calcium caused by LPZ, thus protecting cell viability.

**Conclusions:**

Longterm administration of LPZ induced osteoporotic symptoms in mice, and LPZ triggered calcium increases in osteoblasts in a concentration-dependent manner. Intracellular calcium ([Ca^2+^]_i_) persisted at a high concentration, thereby causing endoplasmic reticulum stress (ERS) and inducing osteoblast apoptosis.

## Introduction

PPIs have long been used as the first-line drugs to treat gastric acid-related diseases by irreversibly binding to H^+^/K^+^-ATPase to inhibit gastric acid secretion (Chen et al. 2016). Generally, PPIs are considered to be well tolerated in clinical practice. However, more than 40% of clinical cases involve irrational drug use, and some patients continue to use PPIs for a long time regardless of the clinical indicators (Grant et al. 2006). To date, the majority of PPIs include omeprazole, lansoprazole, pantoprazole, rabeprazole, esomeprazole and ilaprazole (Ito and Jensen 2010; Gyawali, 2017). In 2010, the FDA warned that patients taking PPIs for more than a year or those taking higher doses may have increased risk of hip, wrist, and spine fractures. Clinical meta-analyses have also shown that PPI use increases the risk of hip, spine, or vertebral fracture (Ito and Jensen 2010; Yu et al. 2011; van der Hoorn et al. 2015; Chen et al. 2016; Poly et al. 2018), and patients treated with PPIs for two years are more likely to have a hip fracture (OR = 1.30, CI = 1.21–1.39) (Corley et al. 2010). In addition, animal experiments showed that pantoprazole treatment for 12 weeks had a negative effect on bone metabolism in young male rats (Matuszewska et al. 2016), and pantoprazole (100 mg/kg/day) could affect fracture healing in mice (Histing et al. 2012; Menger et al. 2020). However, there have been few studies on the molecular mechanism of the effect of PPIs on osteocytes in vitro.

Bone homeostasis is maintained by a balance between osteoclast (OC)-mediated bone resorption and osteoblast (OB)-mediated bone formation mediated (Yin et al. 2019). To date, the literature associated with PPIs and bone loss has focused mainly on the relationship between PPIs and OCs, and few studies have focused on PPIs and OBs (Yuan et al. 2010; Jo 2015). Since new bone formation depends largely on osteoblasts, any factors that promote osteoblast apoptosis could increase the risk of osteoporosis (Guo et al. 2014; Elias and Targownik 2019). In this study, we focused on the of role of OBs in skeletal system injury induced by PPIs.

Naseri et al.showed that both omeprazole and lansoprazole could induce arterial relaxation in a time-dependent manner, and this effect was associated the regulation of intracellular calcium (Naseri and Yenisehirli 2006). Schillinger showed that pantoprazole could affect the uptake of Ca^2+^ in the sarcoplasmic reticulum (SR) by inhibiting SERCA, thus reducing the transient amplitude of calcium and myocardial contractility (Schillinger et al. 2007; Sato et al. 2017). Yurtsever and Aydin hypothesized that omeprazole and lansoprazole may inhibit Rho-kinase, thus affecting Ca^2+^ regulation or blocking calcium channels to inhibit muscle contraction (Aydin et al. 2003; Yurtsever et al. 2011). Aydan (Yenisehirli and Onur 2006) used lansoprazole (100–300 µM) to inhibit Ca^2+^ entry through voltage-gated channels. Thus, it seems that PPIs may have an effect on intracellular calcium homeostasis. In addition, Ca^2+^-ATPase (PMCA), SERCA, Na^+^/K^+^-ATPase, and gastric H^+^/K^+^-ATPase are all P-type ATPases and share high homology with each other (Toyoshima and Cornelius 2013). Furthermore, intracellular calcium overload could significantly induce endoplasmic reticulum stress (Mohsin et al. 2020).

Previous studies have shown that oxidative stress can modulate multiple signaling pathways by activating or inhibiting multiple cytokine and enzyme activities (Zhang et al. 2013). These factors could affect the expression of genes, accelerate apoptosis in cells involved in osteogenesis, including bone mesenchymal stem cells (BMSCs), osteoblasts and osteocytes, and increase the proliferation and differentiation of osteoclasts. This apoptosis results in a decrease in bone resorption relative to bone formation and changes the dynamic balance between osteoclast absorption of bone tissue and osteoblast formation of bone tissue, leading to osteoporosis (Liao et al. 2016; Nie et al. 2019).

ALP, OCN, Runx2, and CoLIα are genes that promote osteoblast differentiation, while Grp78, Caspase-12, Bcl-2, Bax, ATF4, and CHOP are significantly associated with endoplasmic reticulum oxidative stress (Huang et al. 2018; Li et al. 2019; Nagaoka et al. 2019). Thus, we hypothesized that LPZ could inhibit SERCA in OBs, leading to calcium homeostasis disorder. The purpose of this study was to investigate the effect of LPZ on [Ca^2+^]_i_ changes and the viability of MC3T3-E1 cells in vitro and whether LPZ could promote endoplasmic reticulum oxidative stress and induce osteoblast apoptosis. Furthermore, whether LPZ caused OP in mice was examined. The combination of in vitro and in vivo experiments provided a potential mechanism for the deleterious effects of PPIs on the skeletal system.

## Materials and methods

### Animals

ICR mice (SPF grade, 18–22 g, aged 6–8 weeks, half male and half female) were provided by Nanjing Jiangning District Qinglongshan Animal Farm Quality certificate of experimental animals: no. 201824637; production license NO. SCXK (SU) 2017-0-001).

### Drugs

Lansoprazole (purity 99.45%) was provided by Wuhan Yuancheng Co-founder Technology Co., Ltd. Sodium carboxymethyl cellulose (CMC-Na), (viscosity: 600–3000 mPa.s, USP grade) was obtained from Shanghai Macklin Biochemical Co., Ltd.

### Drug administration

The mice were administered lansoprazole orally for 6 months. All mice were placed in separate cages and fed standard laboratory rodent chow and water under standard conditions. During the experiments, the mice were maintained at a constant temperature of 26 ℃ with a 12-h light–dark cycle. After 3 days of adaptive feeding, the mice were randomly divided into three groups: the lansoprazole high-dose group (1000 mg/kg), the lansoprazole low-dose group (250 mg/kg), and the control group (0.5% CMC-Na). The dose was calculated as follows: (unit: ml) = 0.02 × m (unit: g). After the mice were fed for 6 months, the mice in each group were randomly selected to determine the bone mineral density and serum biochemical indices.

### Microcomputed tomography (micro-CT)

Ten mice were randomly selected from each group and sent to the Animal Experimental Center of Nanjing Medical University. After being anesthetized, the mice were subjected to SkyScan1176 in vivo micro-CT scans of the right femur. Using a 12.59 µM pixel size, we used an X-ray at 50 kV and 455 µA to scan the distal metaphysis of the femur. Regions of interest (ROIs) of cancellous bone were taken from layer 50 to layer 100 distant from the growth plate.

### Mouse femur biomechanical examination

Six mice were randomly selected from each group and sacrificed, the complete right femur was collected, and the maximum bone load (maximum breaking force) and the bone structural strength (maximum crushing force) of the femur samples were measured by a YLS-16A small animal bone strength instrument. The apparatus maximum applied pressure was 25 kg, the test bone length range was 20–75 mm, and the minimum reading was 0.001 kg.

### Mouse serum biochemical indicator analysis

After the final administration, peripheral whole blood samples were collected in 1.5 ml plastic centrifuge tubes. Blood samples were centrifuged at 3000 rpm for 10 min, the supernatant was collected, and serum inorganic phosphorus (S-IP), serum calcium (S-Ca) and serum alkaline phosphatase (S-ALP) were measured by an automatic biochemical analyzer.

### Cell culture

MC3T3-E1 cells were cultured in α-MEM medium (Gibco, 11095080) supplemented with 10% fetal bovine serum (FBS) (Biological, 4-001-1ACS), 100 mg/dl glucose, and 1% antibiotics (100 U/ml penicillin G and 100 mg/ml streptomycin) (Gibco, 15140-122) at 37 °C in a humidified atmosphere of 5% CO_2_. The cells were used for future experiments 70–80% confluency.

### Cell viability assay

We chose a dose range of 1–50 µM because in extracellular fluid, the effective concentration of PPIs was relatively low (1–50 µM), which was almost equivalent to the clinical blood concentration of 20–40 mg/dl (Olbe et al. 2003). Pharmacokinetic analysis of clinical proton pump inhibitors showed that the maximum blood concentration of lansoprazole was in the range of 1–15 µM. CCK-8 (Beyotime, C0038) and EdU kits (Beyotime, C0078S) were used to study the effects of PPIs on the proliferation of MC3T3-E1 cells. MC3T3-E1 cells were seeded in 96-well plates (density: 8000 cells/well) until the cells reached ~ 70–80% confluence, after which the cells were treated with LPZ. MC3T3-E1 cells were incubated for 24 h in α-MEM/10% FBS containing different concentrations of LPZ (0, 5, 10, 20, 50 μM), and cells in the vehicle group were cultured in α-MEM/10% FBS containing 0.1% DMSO (v/v) (Sigma, D2650). Then, the cultures were washed with PBS, the medium in each well was replaced with 100 µl of FBS-free α-MEM, and 10% CCK-8 working solution was added. The mixture was incubated for 1 h at 37 °C in a humidified atmosphere of 5% CO_2_ and measured at 450 nm using a microplate reader. The cells in the control group were cultured in α-MEM containing 0.1% DMSO.

### Reverse transcription-quantitative polymerase chain reaction

After being treated with LPZ for 24 h, total RNA was extracted from MC3T3-E1 cells with TRIzol reagent (Thermo Fisher, 15596018). Subsequently, the concentration of RNA in the samples was determined by a TECAN Grating-type multifunctional microplate detector, and the target ratio was between 1.8 and 2.0. Next, RNA was reverse transcribed into cDNA under the following reaction conditions: 37 ℃ for 15 min, 85 ℃ for 5 s, and 4 ℃ for 5 min. Then, the samples were stored at − 20 ℃ for future use. Polymerase chain reaction conditions were set as follows: 95 ℃ for 10 min to predenature the sample; and 95 ℃ for 15 s and 60 ℃ for 1 min to alternate for 40 cycles. GAPDH was used as an internal reference, and the fold change value was used to express the relative gene expression. The primers used in this study are listed in Table 1.

**Table 1:** Primers sequence for PCR

Genes	Forward (5′–3′)	Reverse (3′–5′)
OCN	AGACTCCGGCGCTACCTTGG	CGGTCTTCAAGCCATACTGGTCTG
ALP	TCATTCCCACGTTTTCACATTC	GTTGTTGTGAGCGTAATCTACC
COLΙα	GCTCCTCTTAGGGGCCACT	CCACGTCTCACCATTGGGG
Runx2	ATGCTTCATTCGCCTCACAAA	GCACTCACTGACTCGGTTGG
Caspase-12	TGGCCCATGAATCACATCTAAT	TGGACAAAGCTTCAGTGTATCT
ATF4	TGGCTGGCT GTGGATGG	TCCCGGAGAAGGCATCCT
GAPDH	GGTTGTCTCCTGCGACTTCA	TGGTCCAGGGTTTCTTACTCC
ATP2B1	AGATGGAGCTATTGAGAATCGCA	CCCTGTAACACGGATTTTTCCTT

### Quantification of apoptosis using flow cytometry

Cell apoptosis was examined by Annexin V-FITC and PI staining (Vazyme, A211-02). MC3T3-E1 cells were treated with LPZ (5, 10, 20, 50 µM) for 12 h. After being treated, the cells were washed with precooled PBS twice. The cells were digested with 0.25% trypsin (without EDTA) and then centrifuged, and the centrifuged cells were washed twice with PBS. Then, 500 µl of Annexin V binding solution, 5 µl of Annexin V-FITC staining solution, and 5 µl of propidium iodide (PI) were added to each tube of cells. Cells in the control group were treated with only binding solution but not with staining solution. Double labeling was performed at room temperature for 10 min in the dark before flow cytometric analysis. The cells in the control group were cultured in normal medium containing 0.1% DMSO.

### Cellular calcium analysis

[Ca^2+^]_i_ was measured using the calcium-sensitive fluorescent indicator fluo-3/AM (Keygen Biotech, KGAF023-1). MC3T3-E1 cells were cultured in confocal dishes and loaded with 5 µM fluo-3AM for 30 min in Hank’s balanced salt solution (HBSS) (Gibco, C14175500BT). Then, the cells were washed gently three times with HBSS, incubated with calcium-containing HBSS (Gibco, 24020-133) or calcium-free HBSS and examined by real-time under laser confocal microscopy (Zeiss, LSM 5) within 1 h; recording started when the fluorescence intensity of the cells stabilized. Cells were then promptly treated with LPZ, 2-APB (ApexBio, B6643), ryanodine (ApexBio, B5092), thapsigargin (TG) (Sigma, T9033), verapamil (ApexBio, B1687), and BTP-2 (ApexBio, B7542). Mag-fluo-4/AM (AAT Bioquest, 20401) is a fluorescent probe that labels endoplasmic reticulum Ca^2+^ ([Ca^2+^]_ER_), and Rhod-2/AM (ApexBio, C3276) is a fluorescent probe that labels mitochondrial Ca^2+^ ([Ca^2+^]_mit_) with bright and stable fluorescence. Next, MC3T3-E1 cells were loaded with 2.5 µM Mag-Fluo-4-AM plus 2 µM ER-tracker (Beyotime, C1041) or 5 µM Rhod-2 AM plus MitoTracker green (1 µM) (Beyotime, C1048) for 20 min at room temperature to examine changes in ER and mitochondrial Ca^2+^. Changes in the fluorescence intensity of the indicator represent changes in the Ca^2+^ concentration. In addition, changes in calcium after longterm drug treatment were analyzed by flow cytometry. The cells in the control group were cultured in normal medium containing 0.1% DMSO. The calcium responses were semiquantified by measuring the area under the curve (AUC) using GraphPad Prism 8 (Luptak et al. 2018; Peterson et al. 2019).

### Patch clamp measurement of sodium-calcium exchangers

The electrode was made of hard glass with a microelectrode puller (HEKA). The resistance value of the electrode was 4–8 MΩ. The electrode was filled with water. Single cells with neat edges, no particles on the surface and no contraction are selected. The three-dimensional manipulator was adjusted to move the tip of the electrode to the cell surface, and slight negative pressure was applied. After a high resistance seal formed, fast capacitance was compensated, and negative pressure was applied to break the cell membrane and form a whole cell mark in recording mode. All experiments were carried out at room temperature (25 °C). The stimulation parameters were as follows: constant − 40 mV 250 ms, ramp 60 mV 1000 ms, ramp − 140 mV 2000 ms, ramp − 40 mV 1000 ms, and constant − 40 mV 250 ms. After recording the I_NCX_ current of the cells, the I_NCX_ current was stable for 10 min, and the prepared test drug solution was administered so that the final drug concentration in the cell pool was 0, 10, 50, and 100 µM in turn, which was stable for 20 min after each administration, and the I_NCX_ current of the cells was recorded after the drug diffusion became uniform.

### Western blot analysis

MC3T3-E1 cells were treated with 0.1% DMSO (the vehicle group), 10 µM LPZ, 25 µM 2-APB, and 2 µM TG for 24 h. Whole proteins were extracted using RIPA buffer and then denatured for 10 min at 95 °C. Equal amounts of protein (10 μg) were separated on a 10–12% SDS–PAGE gel and transferred to PVDF (Merck Millipore, ISEQ00010) membranes. The membrane was rinsed and blocked with 5% nonfat milk in TBST at room temperature for 2 h and then incubated overnight at 4 °C with specific primary antibodies against Grp78 (Servicebio, GB11098), CHOP (Cell Signaling, L63F7), Caspase-12 (CST, 2202), Calpain-2 (Abcam, ab126600), cleaved-Caspase-3 (CST, 9661), ATF4 (SAB, 32007), OPG (BTL3466), Rankl (BTL5404T), ALP (Proteintech, 11187-1-AP), OCN (SAB, 23319), GAPDH (SAB, 21612), Actin β (BTL338), Bax (Proteintech, 50599-2-lg), Bcl-2 (ab59348), and Cyt (Servicebio, GB11080). Then, the membranes were washed 3 times for 10 min with TBST and incubated with horseradish peroxidase (HRP)-conjugated anti-rabbit secondary antibodies (Servicebio, GB23303) for 1 h at room temperature. The membranes were washed 3 times for 10 min with TBST, and the blots were visualized with enhanced chemiluminescence reagents and analyzed with ImageJ software.

### Statistical analysis

Measurement data are expressed as the mean ± SD. One-way ANOVA followed by Tukey’s test was used for comparisons among groups, and *P* < 0.05 was considered statistically significant (*P < 0.05, **P < 0.01).

## Results

### Effect of LPZ on bone density, serum biochemical indices and femoral biomechanical properties in mice

**Fig. 1:**
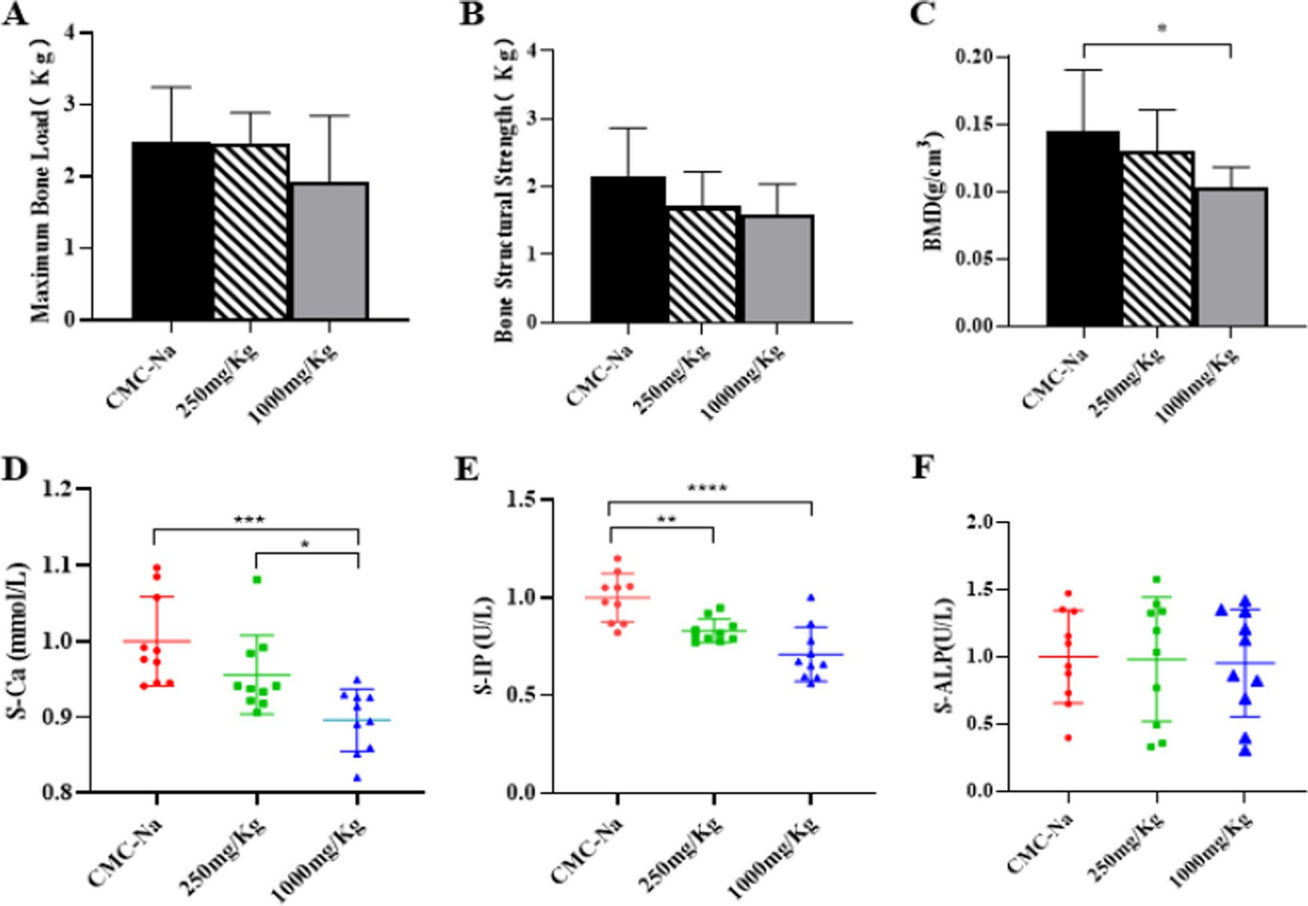
After 6 months of LPZ gavage, the right femurs of male and female mice were scanned by micro-CT to calculate BMD (n = 10); the remaining mice were randomly sacrificed, and blood was collected to examine bone and serum biochemical indicators (n = 10). **A**, **B** Femoral biomechanical strength properties. **C** BMD of the right femur. **D**–**F** Bone serum biochemical parameters

**Fig. 2:**
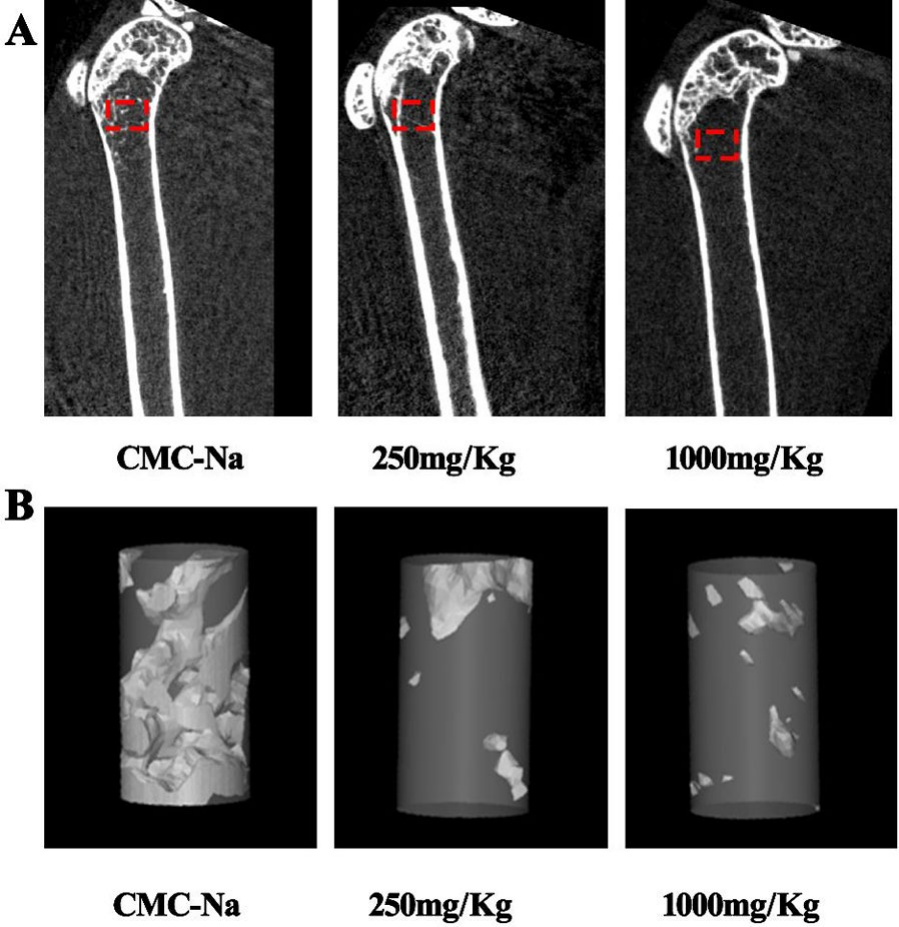
Representative microcomputed tomography (micro-CT) images of the distal femurs. **A** Micro-CT images of femur specimens showed a reduction in trabecular bone microarchitecture after treatment with increasing doses of LPZ (red box). **B** Micro-CT images of cancellous bone structures near the growth plate (n = 7)

After continuous gavage administration for 6 months, there was no significant difference in body weight among the groups. Compared with the CMC-Na group, the low-dose group and high-dose group showed dose-dependent decreasing trends in the material mechanical parameters maximum bone load (maximum breaking force) and bone structure strength (maximum crushing force) in femoral samples (Fig. 1A, B). Furthermore, the low-dose group (250 mg/kg) and high-dose group (1000 mg/kg) showed decreasing trends in femoral BMD, and there was a significant difference between the control group and high-dose group (Fig. 1C). Bone serum biochemical analysis showed that serum ALP (S-ALP) decreased slightly and serum calcium (S-Ca) and serum phosphorus (S-IP) decreased significantly (Fig. 1D–F). Similarly, compared with that in the CMC-Na group, the trabecular bone microarchitecture was thin and loose (Fig. 2).

### Effect of LPZ on MC3T3-E1 cell viability

**Fig. 3:**
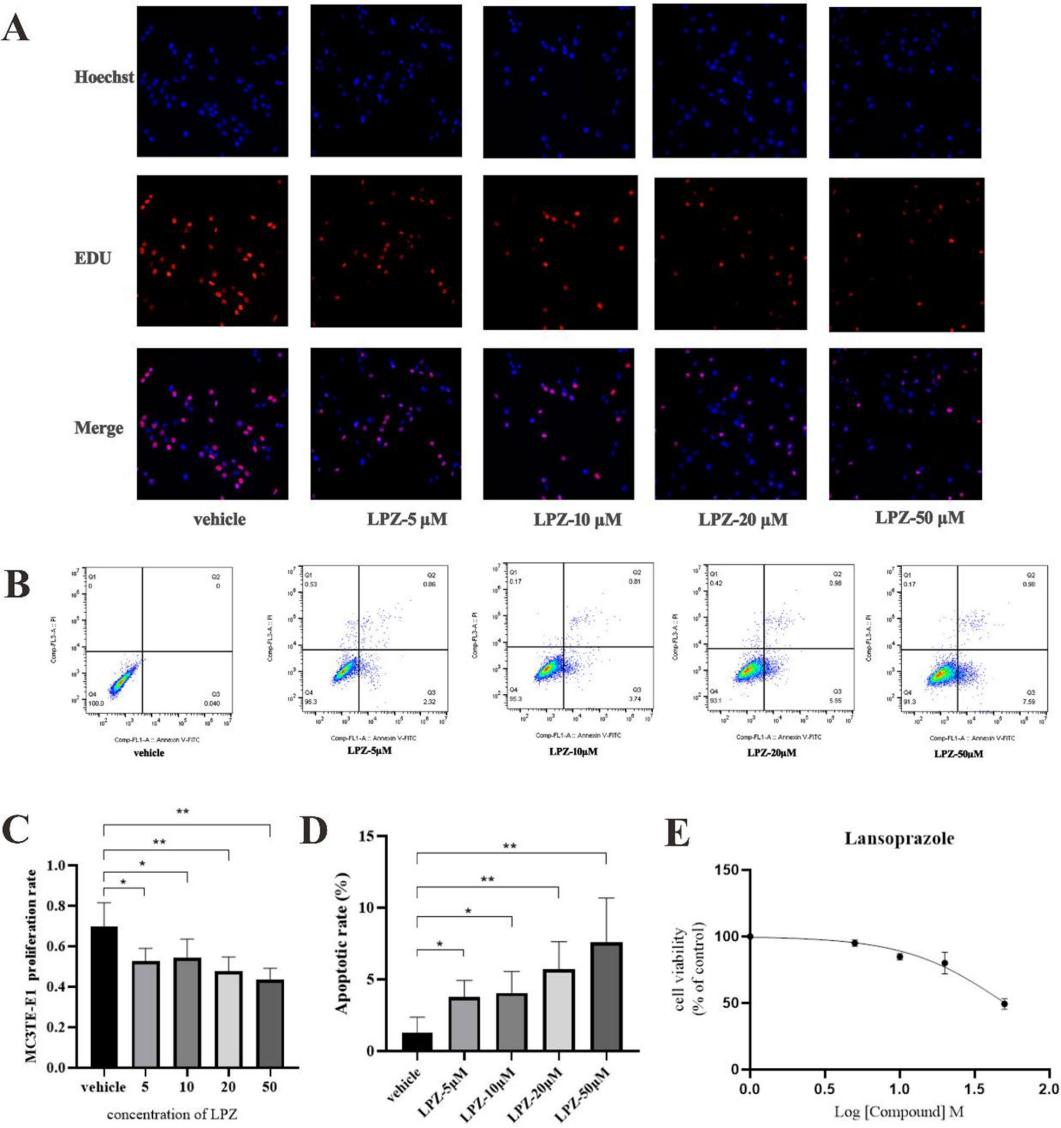
Cell proliferation, viability and apoptosis were examined by CCK-8 assays, EdU assays and flow cytometry. **A** MC3T3-E1 cells were treated with increasing concentrations of LPZ, and the EdU assay showed a reduction in proliferation. **B** Apoptosis in osteoblasts pretreated with LPZ (0, 5, 10, 20, 50 µM) for 24 h. **C** Number of cells, as determined by the EdU assays. **D** Quantitative analysis of apoptotic rates. **E** The viability of MC3T3-E1 cells after LPZ treatment. *P < 0.05, **P < 0.01 (n = 3)

After discovering that LPZ damaged mouse bone tissue, MC3T3-E1 cells, a common osteoblast cell line, was used to explore the mechanism by which LPZ causes bone damage. First, we used EdU assays to determine whether LPZ promoted or inhibited MC3T3-E1 cell proliferation. The proportion of proliferating cells decreased significantly (*P* < 0.05) when cells were treated with LPZ at concentrations of 5, 10, 20, and 50 µΜ (Fig. 3A, C). Next, we further explored whether LPZ could injure MC3T3-E1 cells through an apoptotic mechanism. MC3T3-E1 cells were incubated in α-MEM/10% FBS containing different concentrations (0, 5, 10, 20, and 50 μM) of LPZ. The apoptosis rate in the LPZ treatment group increased in a dose-dependent manner (Fig. 3B, D). Furthermore, LPZ inhibited the viability of MC3T3-E1 cells in a dose-dependent manner, as determined by a CCK-8 assays (Fig. 3E). These results suggested that LPZ inhibited the viability of MC3T3-E1 cells, leading to apoptosis.

### Calcium responses after LPZ treatment in MC3T3-E1 cells

#### LPZ increased calcium through ER Ca^2+^ release

**Fig. 4:**
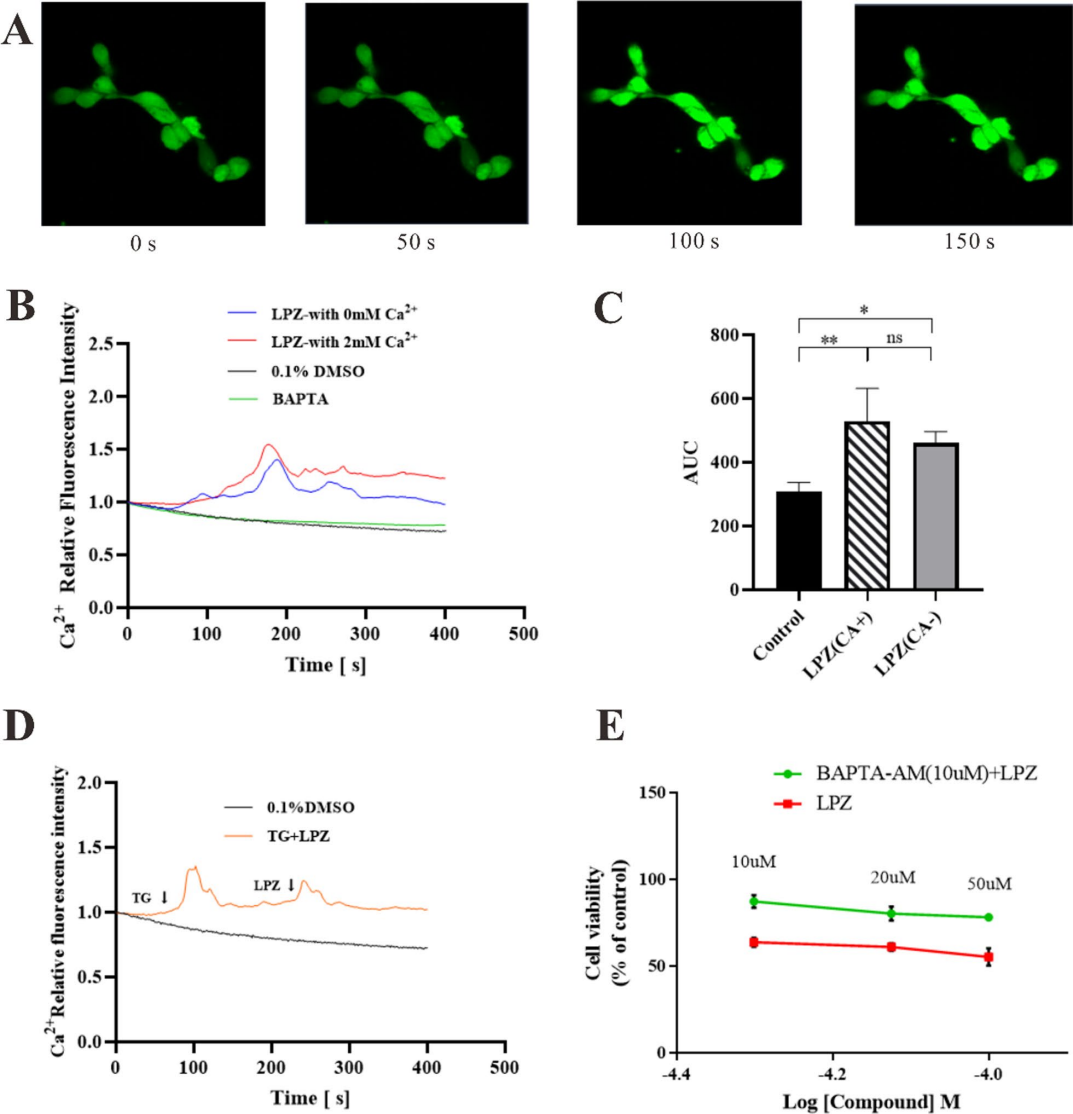
Calcium regulation in MC3T3-E1 cells was observed by confocal microscopy. **A** Representative confocal images (40 ×): After LPZ administration, intracellular calcium fluorescence increased significantly. **B** Quantification of Fluo-3 fluorescence: Ca^2+^ fluorescence was slightly higher with calcium than without calcium. **C** Area under the curve of Fluo-3 fluorescence: There was no significant difference in the area under the curve of calcium fluorescence with or without calcium. **D** Fluo-3 fluorescence after preincubation with TG (n = 3): After preincubation with TG to deplete calcium stores, LPZ did not cause a significant increase in cellular calcium fluorescence. **E** The viability of MC3T3-E1 cells after preincubation with BAPTA/AM and LPZ treatment with CCK-8: BAPTA prevented the increase in Ca^2+^ and could effectively protect cell viability

We used confocal laser scanning microscopy to investigate calcium real-time changes in MC3T3-E1 cells after LPZ treatment. We found that calcium fluorescence significantly increased in MC3T3-E1 cells treated with 50 µM LPZ (Fig. 4A). Moreover, the increase in [Ca^2+^]_i_ was slightly higher in the calcium-containing solution than in calcium-free solution (Fig. 4B). BAPTA is a calcium chelator that can prevent the increase in calcium fluorescence after administration, but there was no significant difference between the two groups (Fig. 4C), which indicated that [Ca^2+^]_i_ originated from both intracellular calcium release and extracellular calcium influx and that intracellular calcium release was mainly required for the LPZ-induced [Ca^2+^]_i_ response in MC3T3-E1 cells. Thapsigargin (TG) is an inhibitor of SERCA with high specificity. MC3T3-E1 cells were pretreated with TG in calcium-free solution to deplete calcium stores in the ER and then treated with 50 µM LPZ, and there was no change in calcium in MC3T3-E1 cells (Fig. 4D). These findings suggested that LPZ-mediated calcium signaling in MC3T3-E1 cells was mainly mediated by ER Ca^2+^ release. In addition, we preincubated the cells with BAPTA/AM to chelate intracellular free Ca^2+^ and found that MC3T3-E1 cell survival was significantly increased (*P* < 0.05). LPZ inhibited the viability of MC3T3-E1 cells; however, the viability of MC3T3-E1 cells that were preincubated with BAPTA/AM was significantly higher than that of MC3T3-E1 cells that were not preincubated with BAPTA/AM, which indicated that the [Ca^2+^]_i_ increase was an important factor in LPZ-induced cell damage and that BAPTA/AM could effectively protect cell viability by blocking this process (Fig. 4E).

#### LPZ-induced calcium increases through the IP3R and SOCE pathways

**Fig. 5:**
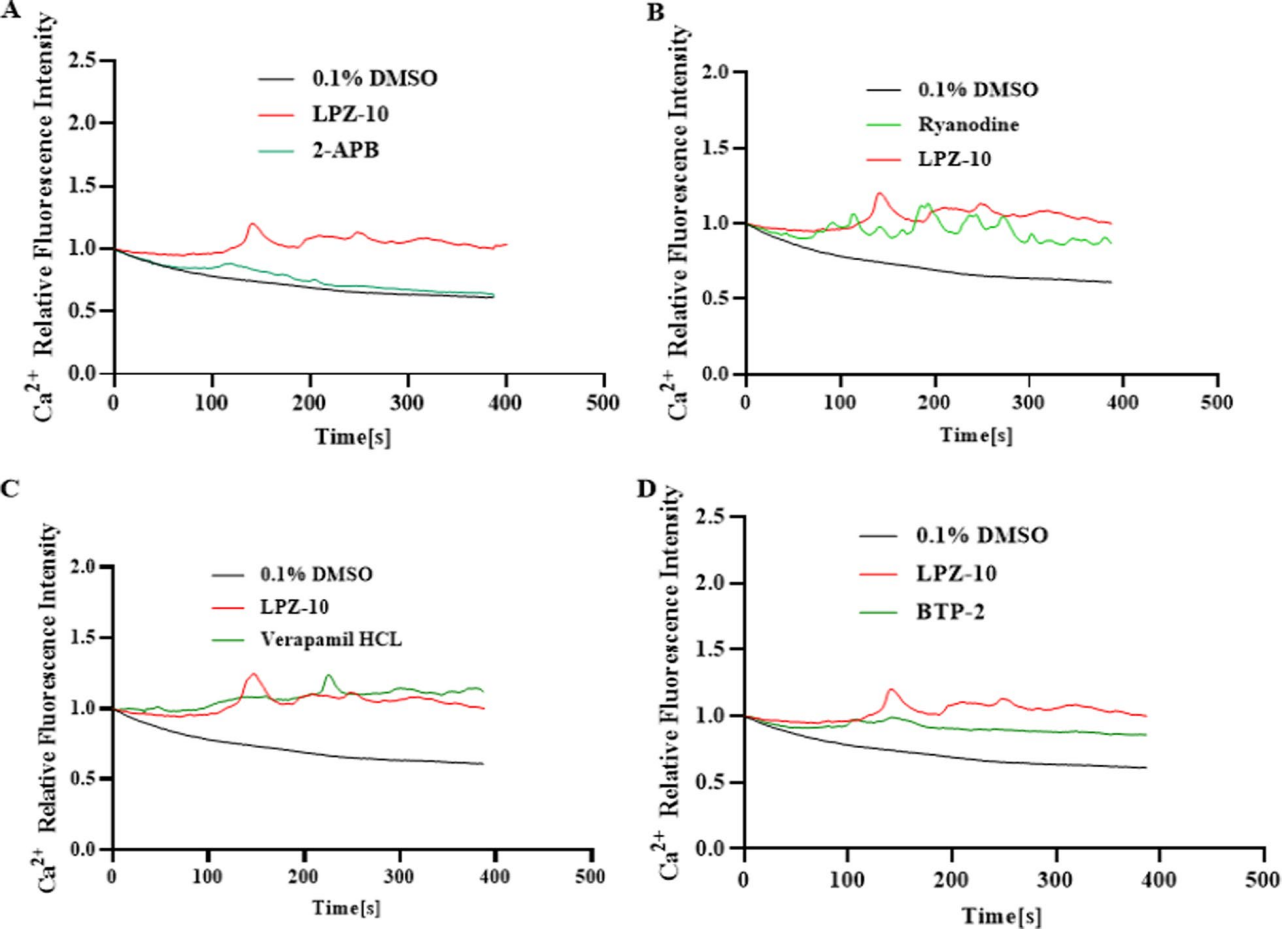
Relative calcium fluorescence change curves. **A** Relative [Ca^2+^]_i_ response in MC3T3-E1 cells that were preincubated with 2-APB in Ca^2+^-free medium. **B** Relative [Ca^2+^]_i_ response in MC3T3-E1 cells that were preincubated with ryanodine in Ca^2+^-free medium. **C** Relative [Ca^2+^]_i_ response in MC3T3-E1 cells that were preincubated with verapamil in Ca^2+^-containing medium. **D** Relative [Ca^2+^]_i_ response in MC3T3-E1 cells that were preincubated with BTP-2 in Ca^2+^-containing medium (n = 3)

Cells were preincubated with 2-APB (25 µM) or ryanodine (20 µM) in calcium-free medium, blocking the IP3R Ca^2+^ release channel or Ryr Ca^2+^ release channel, respectively. Then, the cells were treated with 10 µM LPZ (clinically relevant concentrations). Ryanodine had little effect on [Ca^2+^]_i_ activation by LPZ (Fig. 5B), but 2-APB altered the calcium fluorescence intensity to be consistent with that in the vehicle group (Fig. 5A). In addition, to confirm which calcium channel was responsible for calcium influx, MC3T3-E1 cells were preincubated with verapamil (10 µM), an L-type calcium channel blocker (Jung et al. 2015), or BTP-2 (20 µM), a store-operated calcium (SOC) channel blocker, in calcium-containing medium and then treated with 10 µM LPZ. BTP-2 partly restrained the increase in calcium fluorescence (Fig. 5D); however, verapamil had little effect on [Ca^2+^]_i_ (Fig. 5C). Taken together, these results suggested that LPZ inhibited SERCA in the ER membrane, leading to Ca^2+^ release from the ER through the IP3R channel and promoting the activation of SOCE.

#### LPZ decreased [Ca^2+^]_ER_ and increased [Ca^2+^]_mito_

**Fig. 6:**
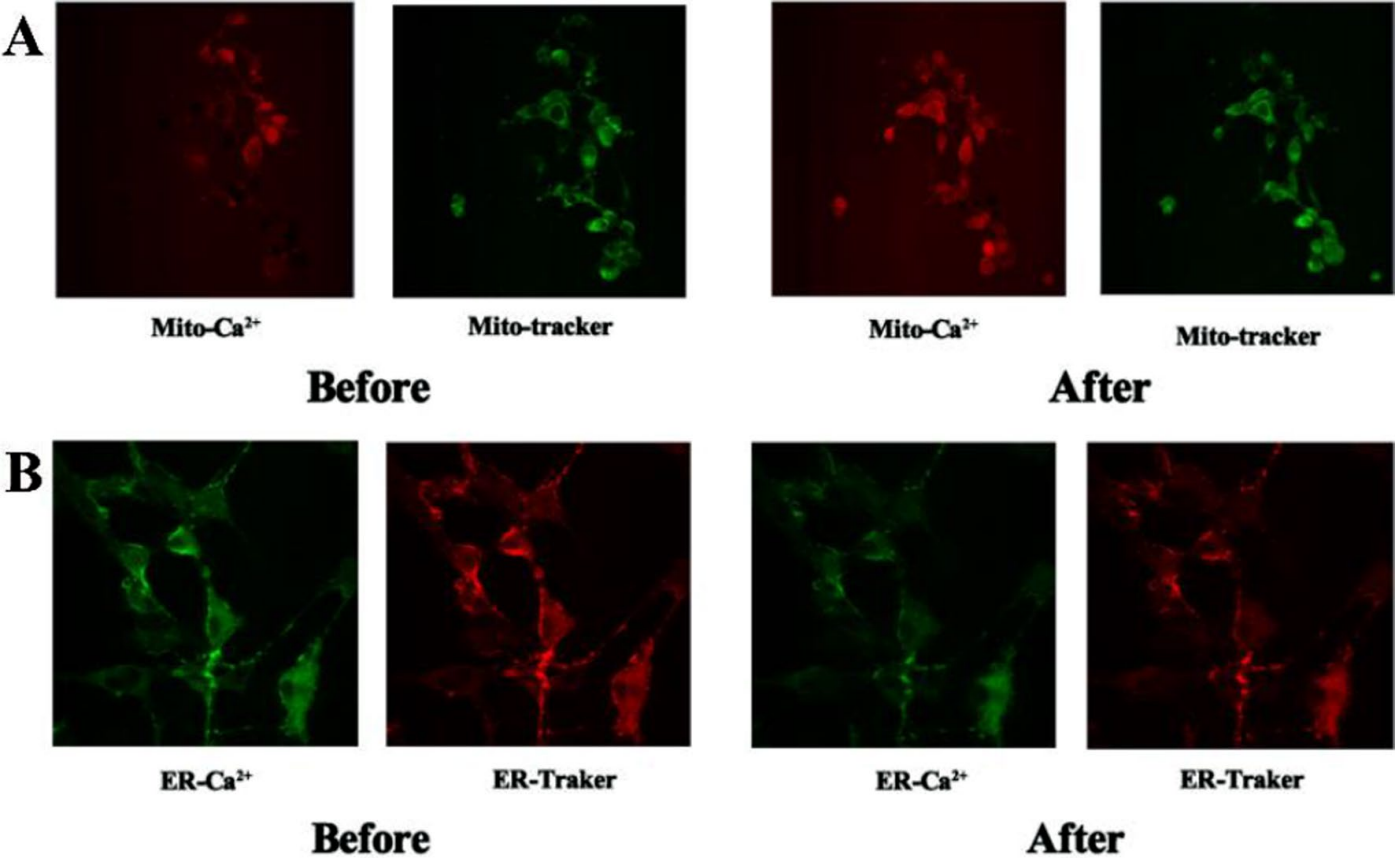
Representative confocal images of MC3T3-E1 cells. **A** MC3T3-E1 cells were double-loaded with ER tracker (red) and Mag-fluo4/AM (green) to determine ER Ca^2+^ levels. **B** MC3T3-E1 cells were double-loaded with MitoTracker Green and Rhod-2AM (red) to determine mitochondrial Ca^2+^ levels. (20×)

Furthermore, to investigate whether LPZ had an effect on the ER and mitochondria, MC3T3-E1 cells were loaded with Mag-fluo4/AM and Rhod-2/AM probes to identify calcium levels in the ER and mitochondria, respectively. As expected, ER Ca^2+^ fluorescence decreased after LPZ (10 µM) was added, while Ca^2+^ in mitochondria slightly increased (Fig. 6). These results indicated that Ca^2+^ was released from the ER and flowed into nearby mitochondria.

### Flow cytometric assessment of calcium

#### Elevated calcium in long-term LPZ-exposed MC3T3-E1 cells

**Fig. 7:**
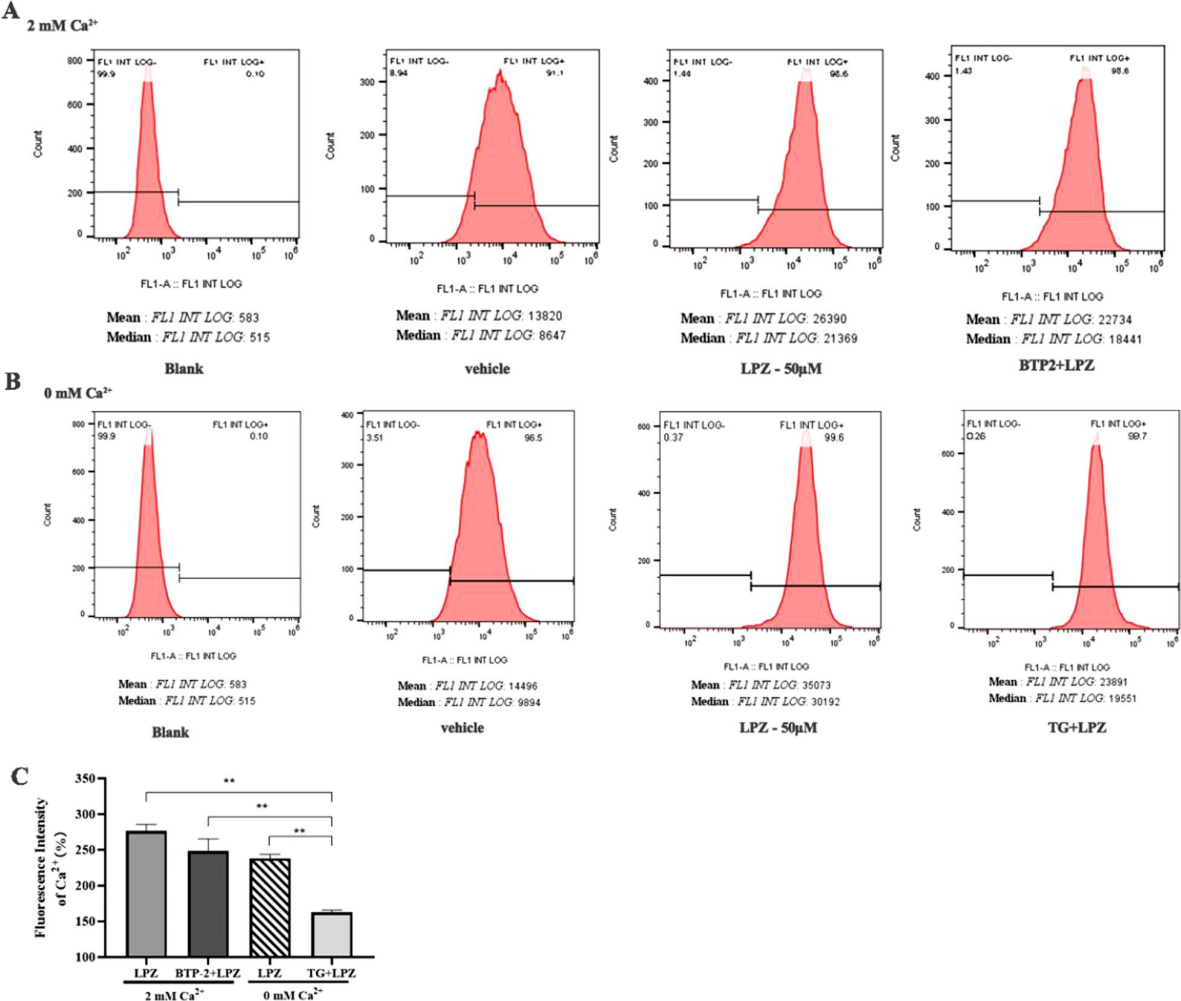
[Ca^2+^]_i_ after longterm treatment with LPZ. **A** [Ca^2+^]_i_ levels in MC3T3-E1 cells that were preincubated with BTP-2 in calcium-containing medium, as detected by flow cytometry. **B** [Ca^2+^]_i_ levels in MC3T3-E1 cells that were preincubated with TG in calcium-free medium, detected by flow cytometry. **C** Quantitative analysis of [Ca^2+^]_i_ levels. (n = 3)

Then, we used flow cytometry to investigate changes in intracellular calcium after longterm treatment (24 h) with LPZ in MC3T3-E1 cells. MC3T3-E1 cells were preincubated with TG (2 μM) in calcium-free medium or preincubated with BTP-2 (20 μM) in calcium-containing medium. Then, LPZ was added to MC3T3-E1 cells and incubated for 1 h. [Ca^2+^]_i_ was significantly higher in calcium-containing or calcium-free medium than in the vehicle group (0.1% DMSO). Furthermore, the magnitude of the calcium increase in MC3T3-E1 cells that were preincubated with BTP-2 in calcium-containing conditions was not significantly different from that in MC3T3-E1 cells that were treated with LPZ in calcium-free conditions. However, taking the vehicle as a reference, the increase in [Ca^2+^]_i_ was significantly lower when MC3T3-E1 cells were preincubated with TG under calcium-free conditions (*P* < 0.01) (Fig. 7). These results were consistent with our confocal microscopy results; the main source of Ca^2+^ was intracellular Ca^2+^, and extracellular Ca^2+^ accounted for only a small amount.

#### [Ca^2+^]_i_ remained high in MC3T3-E1 cells after LPZ exposure

**Fig. 8:**
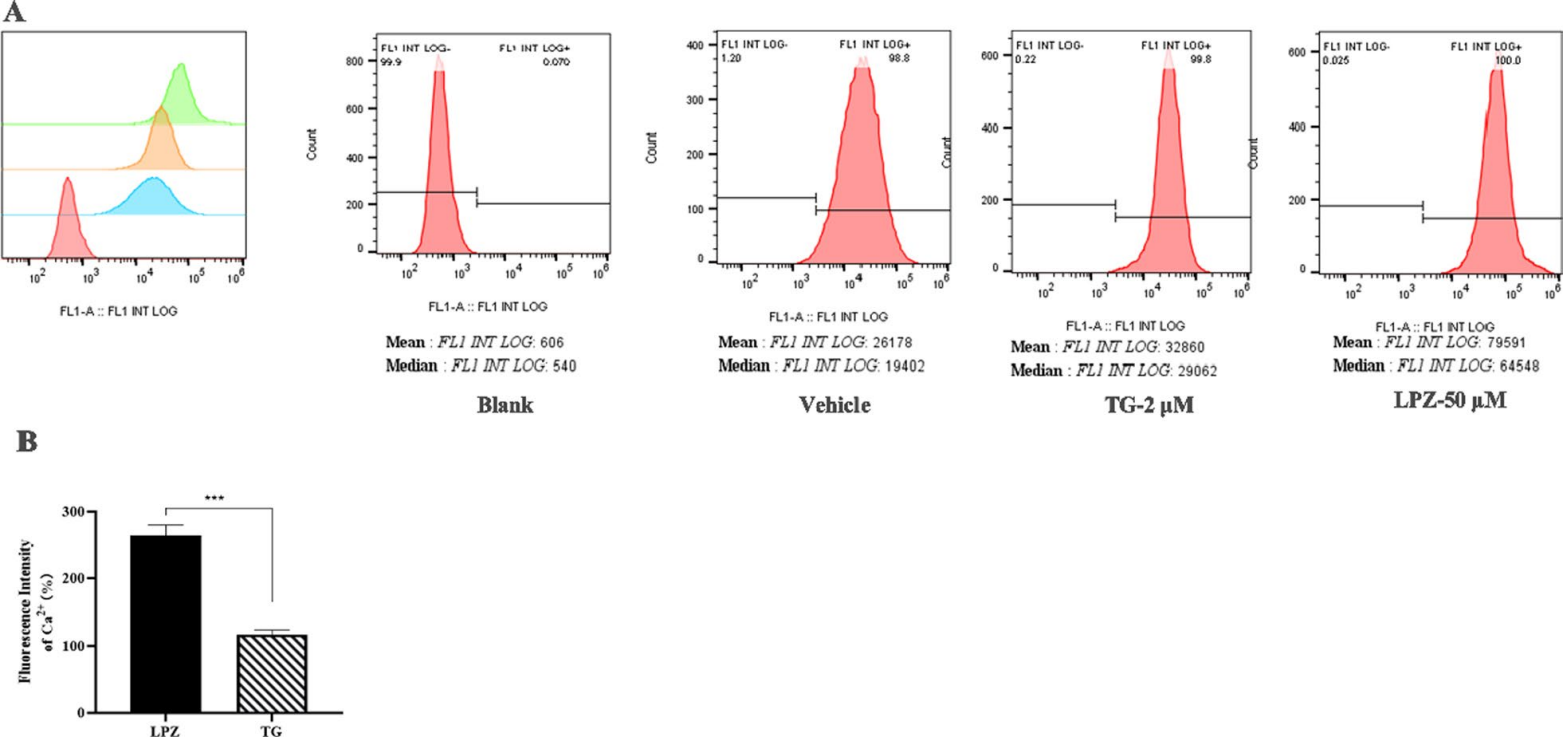
[Ca^2+^]_i_ after longterm treatment with LPZ and TG. **A** [Ca^2+^]_i_ levels in MC3T3-E1 cells that were preincubated with TG in calcium-containing medium, as detected by flow cytometry. **B** Quantitative analysis of [Ca^2+^]_i_ after treatment with LPZ or TG for 24 h (n = 3)

We used TG (2 μM) and LPZ (50 µM) in calcium-containing medium, and incubated the cells for 24 h. The intracellular calcium level remained high in the LPZ group, but the intracellular calcium level was low in the TG group, and there was no significant difference between the TG and vehicle groups. When MC3T3-E1 cells were treated with TG, the [Ca^2+^]_i_ did not remain at a high level for a long time, and Ca^2+^ was expelled by PMCA and NCX on the plasma membrane over time. However, LPZ could maintain [Ca^2+^]_i_ at a high level for a long time, and LPZ might inhibit calcium efflux transporters. These results suggest that LPZ might have an inhibitory effect on SERCA and/or PMCA. Ca^2+^ stress seems to be associated with longterm stress conditions; thus, longterm Ca^2+^ overload may induce apoptosis. We hypothesized that LPZ might inhibit PMCA and/or NCX and that Ca^2+^ cannot be discharged, which explains why the LPZ-treated groups remained calcium overloaded after 24 h (Fig. 8) (n = 3).

### NCX current changes

**Fig. 9:**
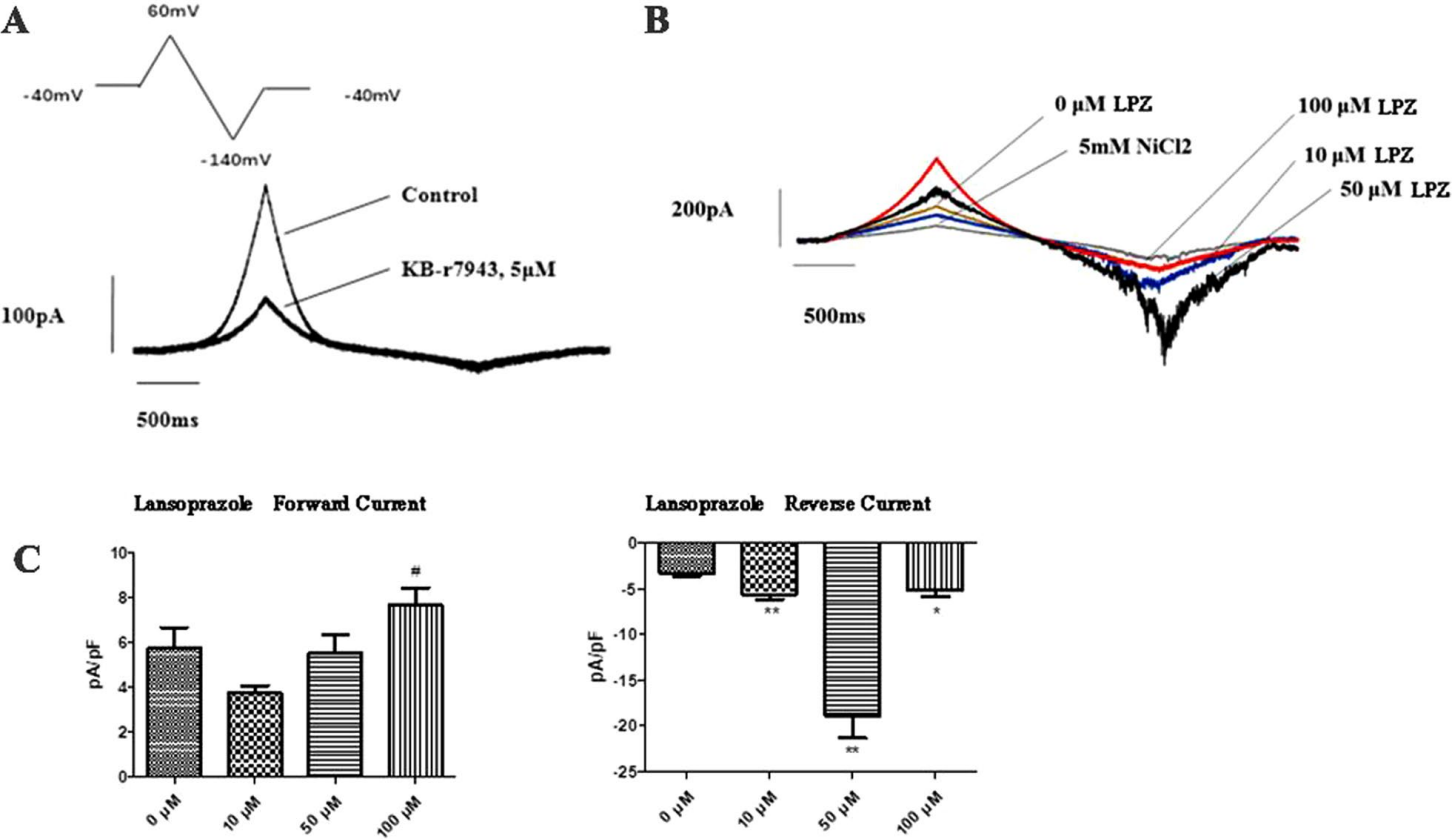
Patch clamp recording of currents. **A** The recorded current was determined to be NCX. **B** Initial induced current of NCX and the effect of different lansoprazole concentrations and Ni^2+^ on I_NCX_; gray: 5 mM NiCl_2_; brown: 0 µM lansoprazole; blue: 10 µM lansoprazole; black: 50 µM lansoprazole; red: 100 µM lansoprazole. **C** NCX current peak statistics of MC3T3-E1 cells in response to different lansoprazole concentrations; forward current density peak statistics, ^#^*P* < 0.05 *vs.* the normal group; reverse current density peak statistics, *P < 0.05 *vs.* the normal group, **P < 0.01 vs*.* normal group. (x + s, n = 6)

In osteoblasts, PMCA and NCX are responsible for calcium balance. We examined the effect of LPZ on NCX transporters using the patch clamp technique. It was shown that 10 µM LPZ increased the reverse current density from − 3.29 ± 1.00 pA/PF (0 µM) to − 5.62 ± 1.46 pA/pF by 70.91%; the current density was -18.9 ± 5.96 pA/pF by 475.10% at 50 µM, and the current density of 100 µM was − 5.21 ± 2.33 pA/pF by 58.52% at 100 µM (Fig. 9). Taken together, the patch clamp results showed that 10, 50, and 100 µM lansoprazole could significantly strengthen the reverse current (forward mode) of the Na^+^-Ca^2+^ exchange current in MC3T3-E1 cells (*P* < 0.05). This result indicated that at concentrations of 10, 50, and 100 µM LPZ, Ca^2+^ could be transported out through Na^+^–Ca^2+^ exchangers. Based on this finding, patch clamp analysis showed that LPZ could concentration-dependently enhance Ca^2+^ transport through Na^+^–Ca^2+^ exchangers in MC3T3-E1 cells. We hypothesized that lansoprazole might stimulate Ca^2+^ overload to prompt an increase in sodium-calcium exchanger activity to maintain a low intracellular calcium level.

### Effects of LPZ on MC3T3-E1 mRNA expression levels

**Fig. 10:**
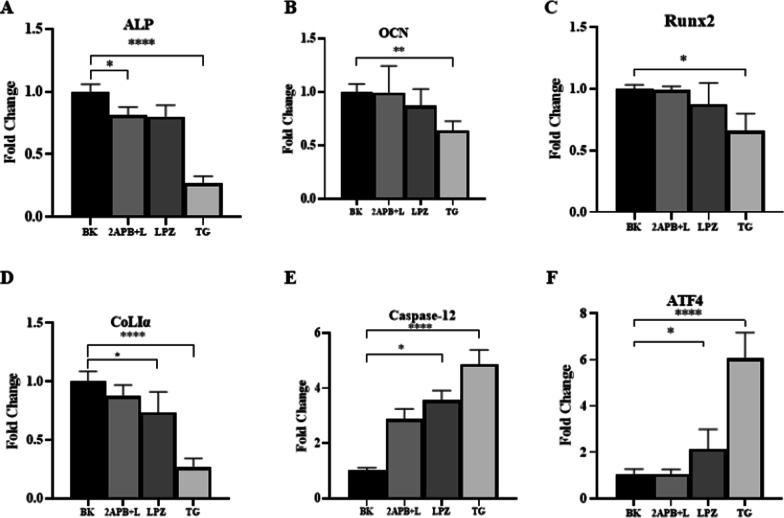
The mRNA expression of OB functional genes and ER stress and apoptosis pathway-related genes (ALP, OCN, Runx2, COLΙα, Caspase-12, ATF4). TG was used as a positive control (n = 3)

MC3T3-E1 cells were treated with 0.1% DMSO (the vehicle group), 10 µM LPZ, 25 µM 2-APB, and 2 µM TG for 24 h, and then we examined the effects of LPZ on genes related to differentiation and the ER stress apoptosis pathway in MC3T3-E1 cells. We found that LPZ could reduce the mRNA expression levels of differentiating and mature MC3T3-E1 cells (ALP, OCN, Runx2, CoLIα) and increase the mRNA expression levels of ER stress markers (Caspase12, ATF4) (Fig. 10).

### Expression of ERS and osteoblast functional protein

**Fig. 11:**
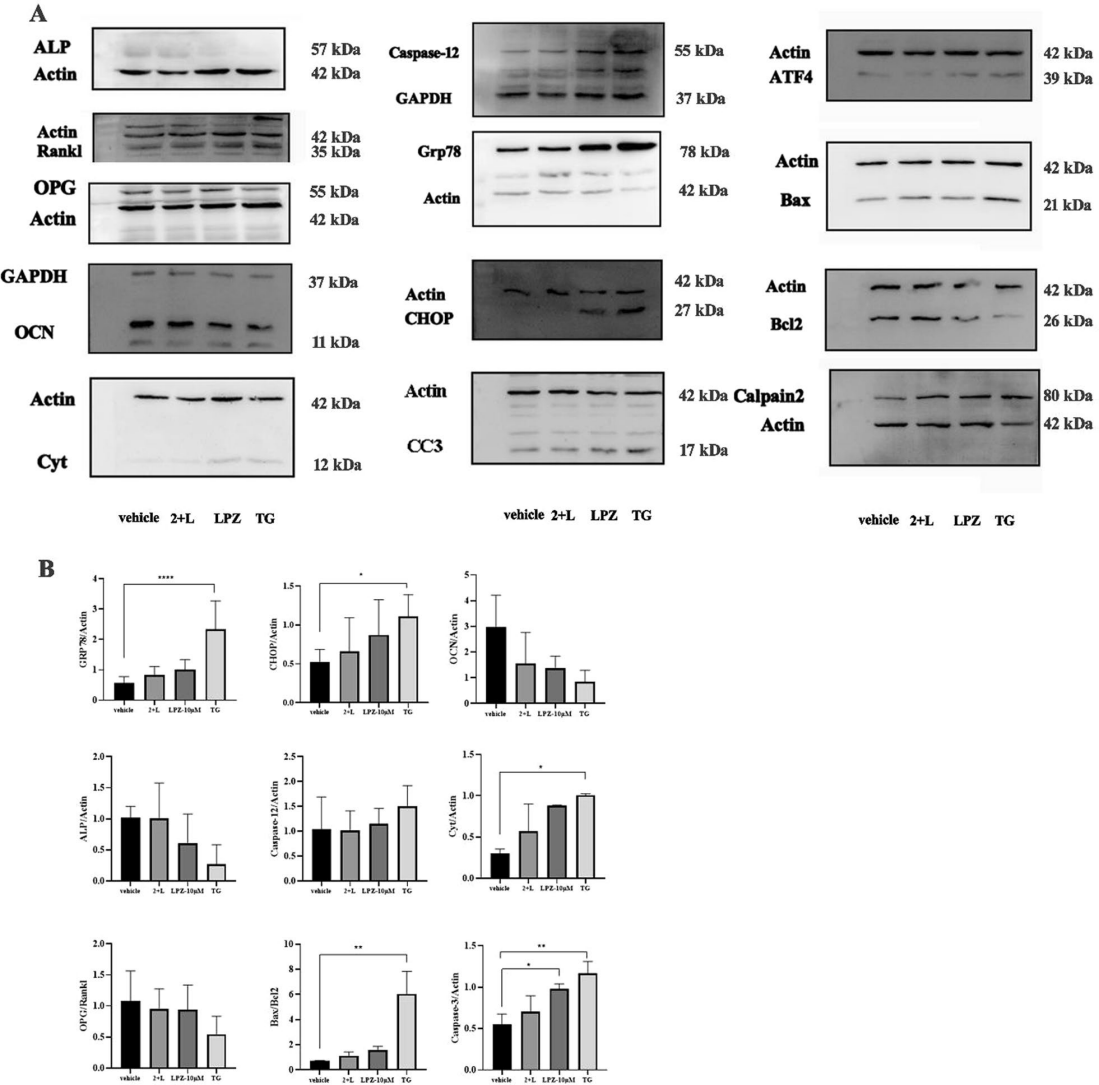
The expression of OB functional genes and ER stress- and apoptosis pathway-related proteins. **A** Western blot analysis after LPZ, 2-APB, and TG treatment. **B** Semiquantitative analysis of protein levels in MC3T3-E1 cells

Previous studies have reported that Caspase-12, GRP78, ATF4, and CHOP are mediators of ERS, Bcl-2 is an antiapoptotic protein, and Bax is a proapoptotic protein (Chiu et al. 2018). TG can induce rapid calcium release from the ER and promote ER stress. We treated the TG group as a positive control group (Chen et al. 2021). In this study, compared with those in the vehicle group, LPZ increased ERS-mediated Caspase-12, Grp78, ATF4, and CHOP protein levels and promoted the expression of cleaved caspase-3, a terminal cleavage enzyme, during apoptosis. However, preincubation with 2-APB reduced ER stress protein expression and abrogated the increase in the Bax/BCL-2 ratio induced by LPZ. It is well documented that Bcl-2 also regulates endoplasmic reticulum calcium homeostasis (Chiu et al. 2018). Moreover, the expression of Calpain-2, a calcium-dependent ER stress protein, was upregulated in the LPZ and TG groups, and 2-APB alleviated the expression of Calpain-2, which meant that LPZ increased intracellular calcium and ER stress. Moreover, the expression levels of MC3T3-E1 osteoblast functional proteins ALP, OPG/Rankl, OCN were reduced in the LPZ group and the TG group compared with the vehicle group, which illustrated that LPZ could induce ERS in MC3T3-E1 cells in a dose-dependent manner, impair the function of MC3T3-E1 osteoblasts, and affect differentiation, secretion, and mineralization, and 2-APB could protect the viability of MC3T3-E1 cells after the treatment of LPZ for 24 h. (Fig. 11).

### Ca-ATPase activity assay

**Fig. 12:**
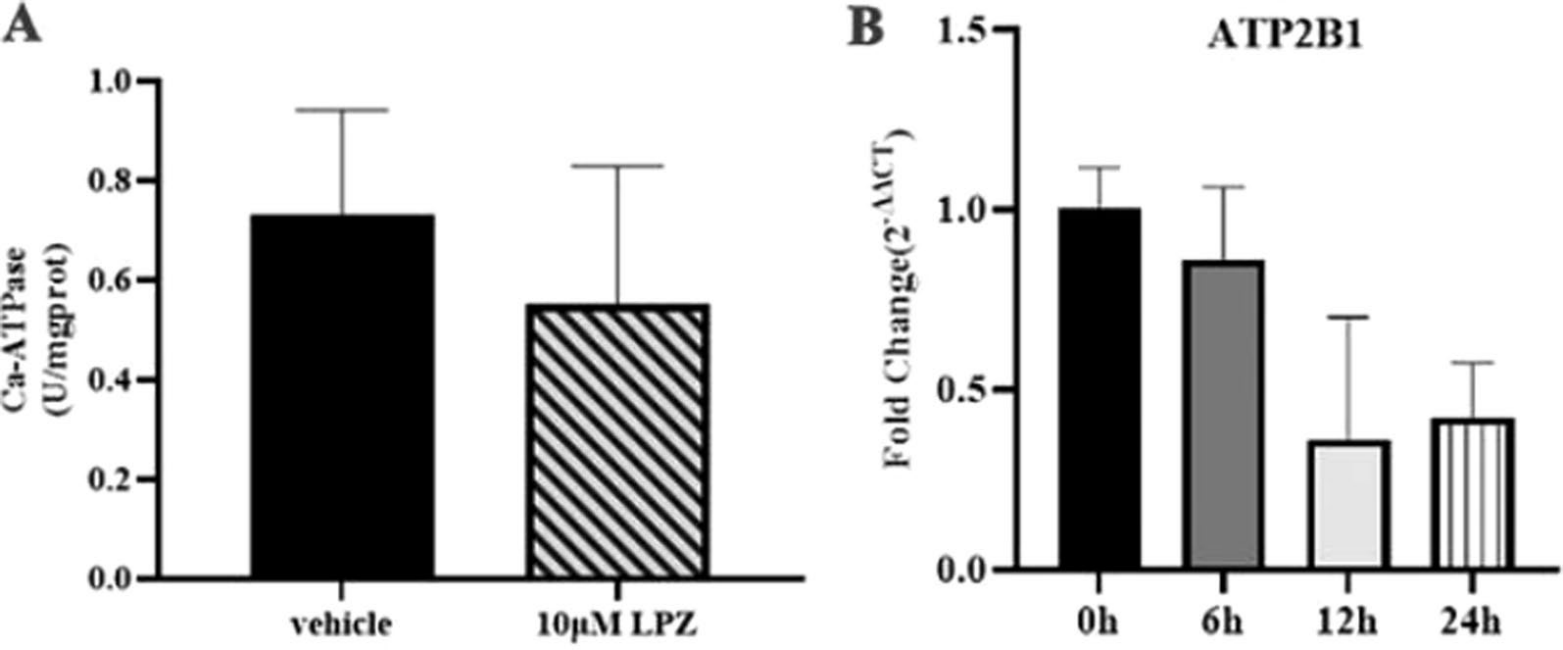
Effects of LPZ on Ca^2+^-ATPase in MC3T3-E1 cells. **A** LPZ reduced Ca^2+^-ATPase activity. **B** LPZ inhibited plasma membrane Ca^2+^-ATPase gene expression (n = 3)

Furthermore, the activity of the Ca-ATP enzyme in MC3T3-E1 cells was examined by phosphorus assays, and the results showed that the activity of the Ca-ATP enzyme was decreased in the LPZ group (Fig. 12). After treatment with 10 μM LPZ, the expression of the Ca^2+^-ATP enzyme (ATP2B1) in the plasma membrane decreased in a time-dependent manner. Although the kit measured the activity of all Ca-ATP enzymes, it also provided some reference for us. These results suggested that LPZ had a negative effect on the calcium pump activity of MC3T3-E1 cells.

### HE staining and immunohistochemical staining in bone

**Fig. 13:**
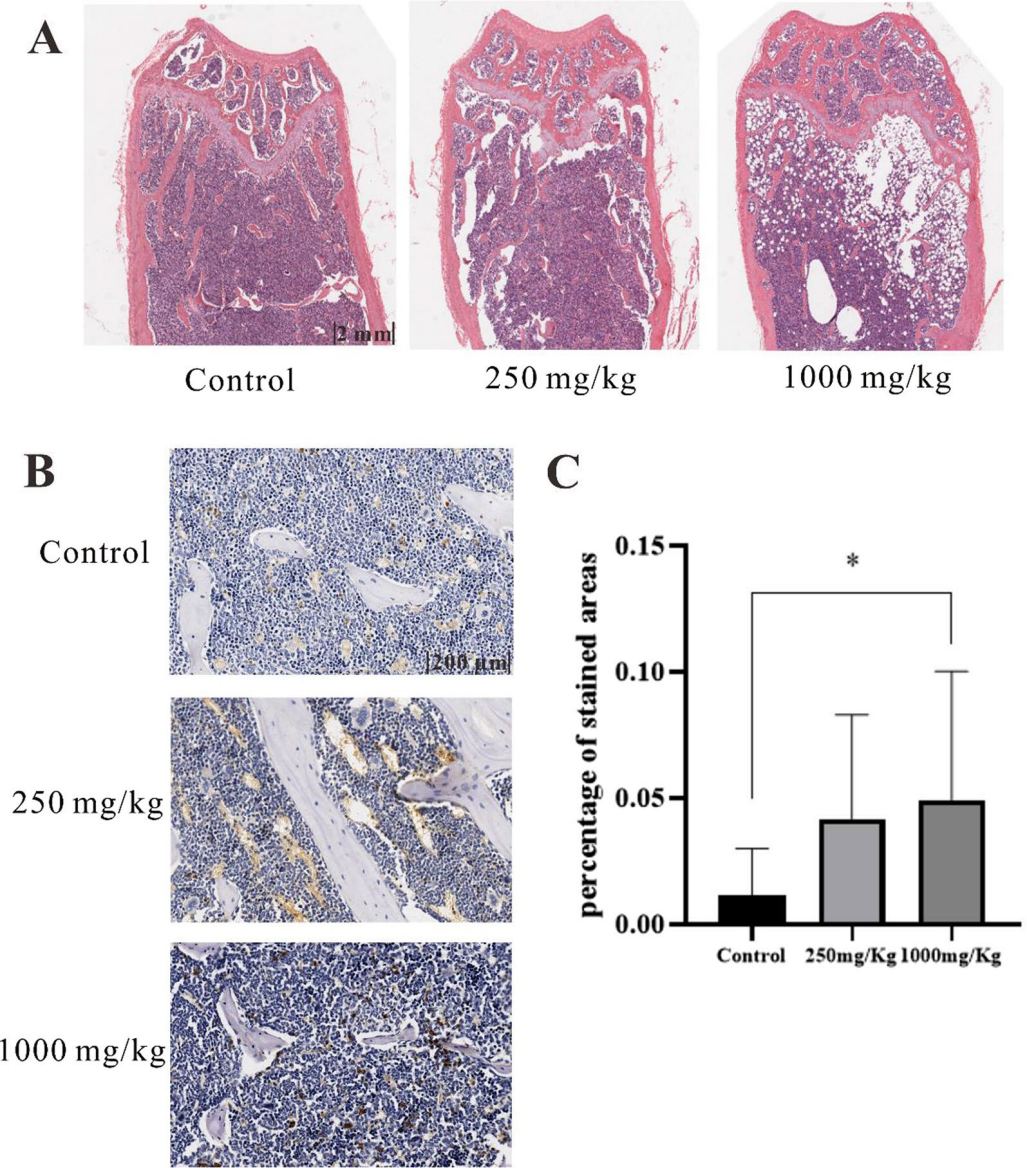
HE staining of femurs and the expression of CHOP after intragastric administration of LPZ for 6 months (n = 3, Scale = 2 mm). **A** Mice treated with increasing doses of LPZ showed bone damage, as determined by HE staining. **B** Mouse femur immunohistochemical analysis of CHOP (n = 3, Scale = 200 µm). **C** Semiquantitative calculation of CHOP was performed with ImageJ

In vivo, HE staining showed that in the control group, the trabecular bone microstructure was dense, with full cancellous bones and few vacuoles. In the low-dose group, trabecular bone was loosely arranged and partially broken, with a decrease in thickness and some vacuoles in the bone marrow. In the high-dose group, trabecular bone was fractured, thinner and had worsened structural integrity, increased separation and more vacuoles in the medulla (Fig. 13A). To examine whether ERS occurred in mice, we used immunohistochemical staining to measure the expression of CHOP, an ER stress marker, in the distal femurs of the different groups. The immunohistochemical results showed that after longterm intragastric administration, the expression of CHOP gradually increased with increasing LPZ doses (*P* < 0.05). It was suggested that longterm LPZ administration may have a negative effect on bone tissue and cause ER stress in the distal femur (metaphysis). Osteoblasts on the trabecular bone surface were also decreased, influencing bone remodeling. This result indicated that endoplasmic reticulum stress occurred in the bone tissues of mice (Fig. 13B, C). In conclusion, the in vivo and in vitro experiments showed that LPZ could lead to increased apoptosis in bone cells and bone injury through endoplasmic reticulum stress.

## Discussion

Here, we hypothesized that LPZ could inhibit the P-type ATPases SERCA and PMCA, leading to an increase in Ca^2+^ in osteoblasts and inducing endoplasmic reticulum stress and apoptosis, and blocking the increase in intracellular calcium had a significant protective effect against osteoblast apoptosis. The increase in osteoblast apoptosis resulted in osteoporosis after 6 months of LPZ administration, which was characterized by an increase in bone microstructure destruction and a decrease in BMD. Therefore, our study suggested that LPZ was a clinical drug, and longterm use of this drug in patients with bone damage may provide a reference for the rational use of such drugs.

In our previous studies, we were able to identify peak plasma concentrations of LPZ in mice that aligned with those observed in response to routine clinical doses (Sun et al. 2017). Prolonged treatment with low and high doses of LPZ led to reductions in BMD and bone structural strength in mice, although there were no changes to overall health, as indicated by consistent body weight during treatment. These results suggested that longterm use of LPZ could influence the balance of bone metabolism and microstructural damage to bone tissue in mice in vivo. The HE staining results showed the morphological structure of trabecular bone in the distal femoral metaphysis (Liu et al. 2017). There was an obvious morphological difference between the control group and the LPZ groups.

Subtle alterations in calcium signaling regulation could cause significant changes in the physiological functions of osteoblasts. Previous studies have shown that changes in calcium can affect the proliferation of MC3T3-E1 cells (Liu et al. 2008; Yu et al. 2018). In vitro, LPZ stimulated Ca^2+^ release in OBs, and BAPTA-AM rescued the increased MC3T3-E1 cell apoptosis triggered by LPZ, suggesting that the increased Ca^2+^ levels participated in LPZ-induced OB apoptosis. Ca^2+^ is an important second messenger, and the precise regulation of Ca^2+^ by calcium pumps, calcium channels, and sodium-calcium exchangers maintains the normal physiological activities of cells (Krebs et al. 2011). Calpain-2 is a Ca^2+^-dependent cysteine protease whose activity and function depend on intracellular Ca^2+^ levels (Bano and Ankarcrona 2018; Wang et al. 2018, 2019). In the present study, LPZ exposure increased the expression of Calpain-2 and Caspase-12 in MC3T3-E1 cells. Caspase-12, which is localized on the cytoplasmic side of the ER membrane, is cleaved and specifically activated during ERS (Zhu et al. 2018; Qiu et al. 2020), illustrating that the increased calcium levels in osteoblasts due to LPZ led to the occurrence of ERS.

Then, we investigated the source of intracellular Ca^2+^ by flow cytometry and confocal microscopy. It was found that 2-APB could significantly abrogate the increase in Ca^2+^ fluorescence in calcium-free medium, and BTP-2 could partly restrain the [Ca^2+^]_i_ increase in calcium-containing medium, suggesting that the [Ca^2+^]_i_ originated from IP3R-mediated ER Ca^2+^ release and that the SOCE pathway could be the main pathway associated with extracellular Ca^2+^ influx. Furthermore, we found a slight increase in the red fluorescence of mitochondria, and the green fluorescence value of the ER decreased. It was suggested that LPZ can release ER calcium into the cytoplasm, causing Ca^2+^ overload, and Ca^2+^ released into the cytosol by the IP3R channel can be taken up by neighboring mitochondria; a similar mechanism was reported by Filadi and Pizzo (2019).

Previous studies have shown that calcium transport significantly affects the progression of excitotoxicity (Bano and Ankarcrona 2018). We hypothesized that LPZ stimulates Ca^2+^ overload to increase sodium-calcium exchanger activity and maintain a low intracellular calcium balance (Sosnoski and Gay 2008; Bano and Ankarcrona 2018). PMCA is responsible for calcium efflux in parallel with NCX, and promotion of calcium efflux by NCX might indirectly reflect Ca^2+^ overload. Furthermore, Liu-zhong Wu et al. found that increased Ca^2+^ signaling significantly promoted the differentiation of bone marrow mesenchymal stem cells into osteoblasts in the presence of insulin-like growth factor 1. Osteoblast marker protein expression was significantly increased, and the expression of SERCA and IP3R was also significantly increased. In conclusion, calcium signaling plays an important role in every stage of bone cells, and Ca^2+^ might not only promote differentiation in bone marrow stem cells but also stimulate apoptosis in osteoblasts (Wu et al. 2020).

Previous studies have confirmed that ER stress plays a key role in the pathogenesis of disuse osteoporosis (Wu et al. 2014; Li et al. 2017). In this study, we also observed increased CHOP expression in mouse tissue in relation to LPZ dose. In addition, the expression of ER stress-related proteins and osteoblast functional proteins was measured by Western blotting. MC3T3-E1 cells were preincubated with 2-APB (20 µM) to confirm the main pathway associated with the increase in [Ca^2+^]_i_. 2-APB has independent targets, including IP3R-dependent ER Ca^2+^ release and store-operated Ca^2+^ (SOC) channels in osteoblasts. In this study, we found that LPZ promoted the activation of caspase 12, Bax/Bcl-2, and caspase-3 induced by ER stress (Wu and Kaufman 2006; Sato et al. 2015; Pihan et al. 2017). Then, we examined some ER stress markers, including Bip/Grp78, ATF4, and CHOP (Nakamura et al. 2013). We found that LPZ increased the expression of Grp78 and CHOP in MC3T3-E1 cells, which indicated that LPZ caused ER stress in osteoblasts. However, when cells were preincubated with 2-APB, the expression of Grp78, CHOP, caspase-12, Bax/Bcl-2, and caspase-3 induced by LPZ was suppressed. Ca^2+^ release from the ER through the IP3R channel and Ca^2+^ influx via the SOCE pathway contributed to the development of ER stress in osteoblasts (Zhang et al. 2020). Additionally, LPZ treatment was associated with a reduction in the ratio of OPG/Rankl, which has been previously shown to be an indication of bone formation or bone resorption (Tantikanlayaporn et al. 2020). We hypothesized that LPZ may destroy the dynamic balance between OB bone formation and OC bone resorption, stimulating bone resorption rather than bone formation. Some animal experiments were consistent with our results and showed that different kinds of PPIs can cause a decrease in the OPG/Rankl ratio at both the cell and animal levels (Fossmark et al. 2012; Hoff et al. 2020). 2-APB rescued the damage to MC3T3-E1 cells induced by LPZ, and the expression of the osteoblastic differentiation markers ALP and OCN was increased (Chou et al. 2005). Calcium signaling is important in bone remodeling and bone healing, and imbalanced Ca^2+^ homeostasis can affect osteoblast activity and further affect bone formation (Han et al. 2018). Furthermore, we noticed that LPZ treatment enhanced the expression of CytC, suggesting that mitochondrial dysfunction might be a possible mechanism by which LPZ induces apoptosis in MC3T3-E1 cells.

Of course, there are some shortcomings in our research. We only investigated the effects of LPZ on osteoblasts but not osteoclasts. In addition, our study only used MC3T3-E1 cells, and no primary cells were used to confirm the changes in related indicators. Overall, our results highlighted the possible mechanisms of LPZ-induced damage to osteoblasts and the significant effects of longterm administration of LPZ on the skeletal system.

## Conclusions

**Fig. 14:**
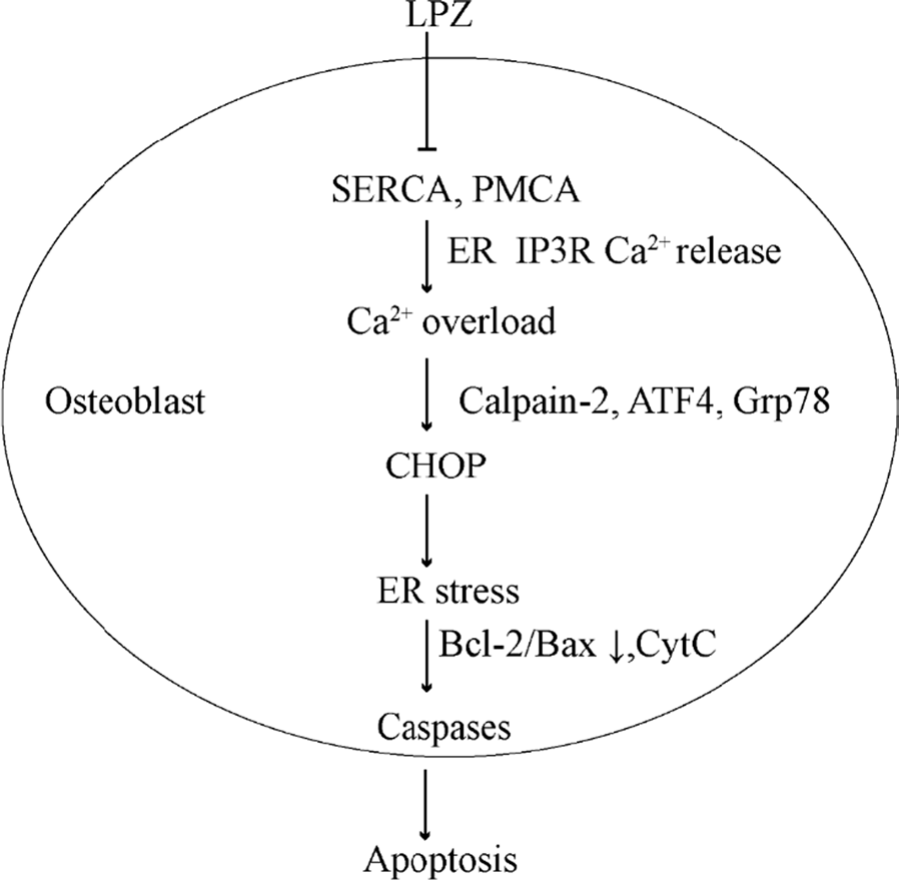
Possible mechanisms underlying the changes in [Ca^2+^]_i_

This study was the first to explore LPZ-induced osteoporosis at the local level using changes in [Ca^2+^]_i_ as an entry point. LPZ inhibited MC3T3-E1 cell viability and triggered significant decreases in BMD and S-Ca, and S-IP levels in mice, inducing osteoporotic symptoms. LPZ could cause persistent increases in the [Ca^2+^]_i_ mediated by the IP3R and SOCE pathways and promote ER stress by inhibiting PMCA and SERCA, thus promoting apoptosis and ultimately causing a decrease in MC3T3-E1 cell viability and function (Fig. 14). PPIs could disturb Ca^2+^ homeostasis in the ER, increase [Ca^2+^]_I_, and activate the Calpain-2/Caspase-12 and Grp78/ATF4/CHOP pathways, ultimately triggering osteoporosis.

LPZ could enter MC3T3-E1 cells through the cell membrane and inhibit SERCA and PMCA. Ca^2+^ was released from the endoplasmic reticulum and maintained at a high level, causing calcium toxicity and triggering the endoplasmic reticulum and mitochondrial apoptotic pathways, ultimately leading to a decline in osteoblast activity and function.

## Data Availability

Data are available upon reasonable request. The data used in the current study are available from the corresponding author on reasonable request.

## Abbreviations

PPIs: Proton pump inhibitors
HE: Hematoxylin–Eosin
LPZ: Lansoprazole
OB: Osteoblast
OC: Osteoclast
BMD: Bone mineral density
PMCA: Plasma membrane Ca^2^^+^-ATPase
SERCA: Sarcoplasmic reticulum Ca^2^^+^-ATPase
TG: Thapsigargin
S-IP: Serum inorganic phosphorus
S-Ca: Serum calcium
S-ALP: Serum alkaline phosphatase
BMD: Bone mineral density
[Ca^2^^+^]_i_: Intracellular calcium
ERS: Endoplasmic reticulum stress
OC: Osteoclasts
OB: Osteoblasts
BMSCs: Bone mesenchymal stem cells
Grp78: Glucose regulated proteins78
CHOP: C/EBP-homologous protein
ATF4: Activating transcription factor 4
OPG: Osteoprotegerin
ALP: Alkaline phosphatase
OCN: Osteocalcin
GAPDH: Glyceraldehyde-3-phosphate dehydrogenase
Runx2: Runt related transcription factor 2
COLIα: Collagen type I alpha
